# E-Screening for Prenatal Depression in Kampala, Uganda Using the Edinburgh Postnatal Depression Scale: Survey Results

**DOI:** 10.2196/51602

**Published:** 2025-01-14

**Authors:** Hasifah Kasujja Namatovu, Mark Abraham Magumba, Dickens Akena

**Affiliations:** 1 Department of Information Systems, College of Computing and Information Sciences, Makerere University Kampala, Makerere University, P.O Box 7062, Kampala, Uganda, 256 774049030; 2 Department of Psychiatry, Makerere University College of Health Sciences, Kampala, Uganda

**Keywords:** perinatal, prenatal, antenatal, antepartum, depression, Edinburgh Postnatal Depression Scale

## Abstract

**Background:**

Perinatal depression remains a substantial public health challenge, often overlooked or incorrectly diagnosed in numerous low-income nations.

**Objective:**

The goal of this study was to establish statistical baselines for the prevalence of perinatal depression in Kampala and understand its relationship with key demographic variables.

**Methods:**

We employed an Android-based implementation of the Edinburgh Postnatal Depression Scale (EPDS) to survey 12,913 women recruited from 7 government health facilities located in Kampala, Uganda. We used the standard EPDS cutoff, which classifies women with total scores above 13 as possibly depressed and those below 13 as not depressed. The *χ*^2^ test of independence was used to determine the most influential categorical variables. We further analyzed the most influential categorical variable using odds ratios. For continuous variables such as age and the weeks of gestation, we performed a simple correlation analysis.

**Results:**

We found that 21.5% (2783/12,913, 95% CI 20.8%‐22.3%) were possibly depressed. Respondents’ relationship category was found to be the most influential variable (*χ*^2^_1_=806.9, P<.001; Cramer’s V=0.25), indicating a small effect size. Among quantitative variables, we found a weak negative correlation between respondents’ age and the total EPDS score (*r*=−0.11, *P*<.001). Similarly, a weak negative correlation was also observed between the total EPDS score and the number of previous children of the respondent (*r*=−0.07, *P*<.001). Moreover, a weak positive correlation was noted between weeks of gestation and the total EPDS score *(r*=0.02, *P*=.05)

**Conclusions:**

This study shows that demographic factors such as spousal employment category, age, and relationship status have an influence on the respondents’ EPDS scores. These variables may serve as proxies for latent factors such as financial stability and emotional support.

## Introduction

### Background

New and expectant mothers face several unique health challenges related to the physical and psychological changes accompanying motherhood. However, in low-income settings where mothers grapple with meeting basic physical needs such as access to safe water, proper nutrition, health care facilities, and health workers, psychological needs may end up being neglected. These needs are important factors as they can impact the physical health of the mother and the development of the child [1], and have been reported to increase the risk of stress, anxiety, and depression [2]. For pregnant women, these needs range from receiving affection and support throughout pregnancy, especially from spouses or partners, friends, and family [2]. Listening to their concerns, sharing resourceful information, and offering assistance are additional psychological needs of a pregnant woman [3]. Unmet needs, along with poverty, lack of spousal or partner support, early and unplanned pregnancies, and physical or verbal abuse, have accelerated perinatal depression [4-7]. Perinatal depression, that is depression that occurs during pregnancy, around childbirth, or within the first year post partum, affects households worldwide. It often co-occurs with other medical or mental health illnesses and frequently goes undetected and untreated [8,9]. A study conducted by Fisher et al [10] reported that approximately 16% and 20% of women in low- and middle-income countries experienced antenatal and postnatal depression, respectively, with variations across settings.

Major depressive disorder in mothers affects 6% to 17% of pregnancies worldwide and can lead to negative outcomes such as preterm delivery, low birth weight [11,12], poor cognitive outcomes, and psychiatric morbidity in childhood and adolescence [13]. Furthermore, it affects a mother’s ability to manage her children’s feeding practices, contributes to poor socio-emotional development, and increases the likelihood of disruptive behavior [14,15]. In severe antenatal cases, perinatal depression may lead to suicidal ideation if left untreated [7,16]. A recent Lancet series highlighted the increasing global burden of mental health disorders, including maternal depression [17]. Although it is the most commonly diagnosed complication of childbirth, there are widespread gaps in screening, detection, and treatment of women affected by this incapacitating condition [18].

In Uganda, the prevalence of postnatal depression has been estimated to be 27% among patients attending primary care clinics [19], and yet it remains largely neglected, similar to other psychosocial disorders. The diagnosis of depressive illness is challenging and is sometimes prone to misdiagnosis [20]. This is largely attributed to (1) a lack of experience and knowledge of the professionals involved, (2) the complexity of clinical presentation [21], and (3) not consistently applying the routine diagnostic criteria during the initial evaluation process [22]. In Uganda, there are no formalized structures for depression screening during antenatal and postnatal visits, leaving individuals to self-diagnose, recognize their need for specialized assistance, and independently locate therapists. This is a significant challenge, considering some symptoms of depression may be attributed to other ailments, suggesting that afflicted individuals may even fail to effectively communicate their situation.

The World Health Organization recommends the integration of perinatal mental health care into primary care. However, in Uganda, this integration is not possible due to the vertical approach to service design, wherein maternal and mental health services are provided separately [23]. The goal of this paper is to establish statistical baselines for the prevalence of prenatal depression in Kampala and understand its relationship with key demographic variables.

### Related Work

There have been several previous studies on maternal depression in Uganda and other sub-Saharan countries using a wide array of tools. For instance, Muhwezi et al [24] validated the 4-item subjective well-being subscale derived from the COMPASS OP Treatment Assessment System [25] in Uganda. Spies et al [26] and Baggaley et al [27] employed the 6-item and 10-item Kessler Screening Scale for Psychological Distress in South Africa and Burkina Faso, respectively. Kaaya et al [28] used the Hopkins Symptom Checklist-25, derived from the 90-item Symptom Checklist [29] in Tanzania. Kushniruk et al [30] employed the Centre for Epidemiological Studies Depression scale [28] in both Tanzania and South Africa. Chibanda et al [31] validated the Edinburgh Postnatal Depression Scale (EPDS) [32] in Zimbabwe. Two broad approaches are usually employed for tool validation, namely a psychiatrist’s evaluation or comparison with gold standard tools. All the above studies except that by Baggaley et al [27] employed the Mini-International Neuropsychiatric Interview tool for validation [33]. Baggaley et al [27], considered a psychiatrist’s assessment as the gold standard. Other tools mentioned in the literature, although not necessarily used in sub-Saharan Africa include the 9-item Patient Health Questionnaire, 20-item Self-Report Questionnaire, and Visual Analogue Scale. However, the EPDS is more widely applied, particularly for maternal depression screening.

The vast majority of studies are geared toward tool validation. Although tool validation is a component of our study, the primary objective is to establish statistical baselines for antenatal maternal depression in Kampala, Uganda; therefore, we employed a comparatively large number of respondents. The data comprises 12,913 records of EPDS results collected from pregnant women attending antenatal clinics in 7 health facilities in Kampala namely: Kitebi Health Centre HCIV, Kawempe Mulago Referral Hospital, Kampala Capital City Authority (KCCA) Health Centre HCIII, Bugolobi Health Centre HCIV, Mengo Kisenyi Health Centre HCIV, Kasubi Kawaala Health Centre HCIII, Komamboga Health Centre HCIII, Kasangati Health Centre HCIII. The data was collected between January 2022 and April 2022.

## Methods

### Ethical Considerations

This study was approved by the Research Ethics Committee of Mulago Hospital (MHREC 2021-57) and the Uganda National Council for Science and Technology (SS945ES). Additional approvals were obtained from KCCA as required for research conducted in KCCA facilities (DPHE/KCCA/1301). Written consent was obtained from all participants for participation and publication of the findings. The data were deidentified. Each respondent was given financial compensation of USh 20,0000 (US $5)

### Study Design

The study participants were expectant mothers receiving antenatal care. The data was collected by 17 research assistants using an Android-based implementation of the EPDS. The results were then transmitted to a remote MySQL server instance via an internet connection. The respondents were required to have signed a consent form. In addition to the 10-question EPDS tool, they completed a general questionnaire that collected basic demographic data and contact information for potential follow-up, for instance, during the validation phase. This questionnaire was also administered in an electronic form via the Android platform.

### Study Setting

In Kampala, there are a total of 1458 health facilities, comprising 26 government-owned, 1371 private-for-profit, and 61 private-not-for-profit establishments [34]. Of these, 11 government-owned facilities provide antenatal care services to the public. In this study, we focused on 7 of the 26 government-owned facilities.

### Data Collection Instrument (Android Implementation of EPDS)

**Figure 1.**
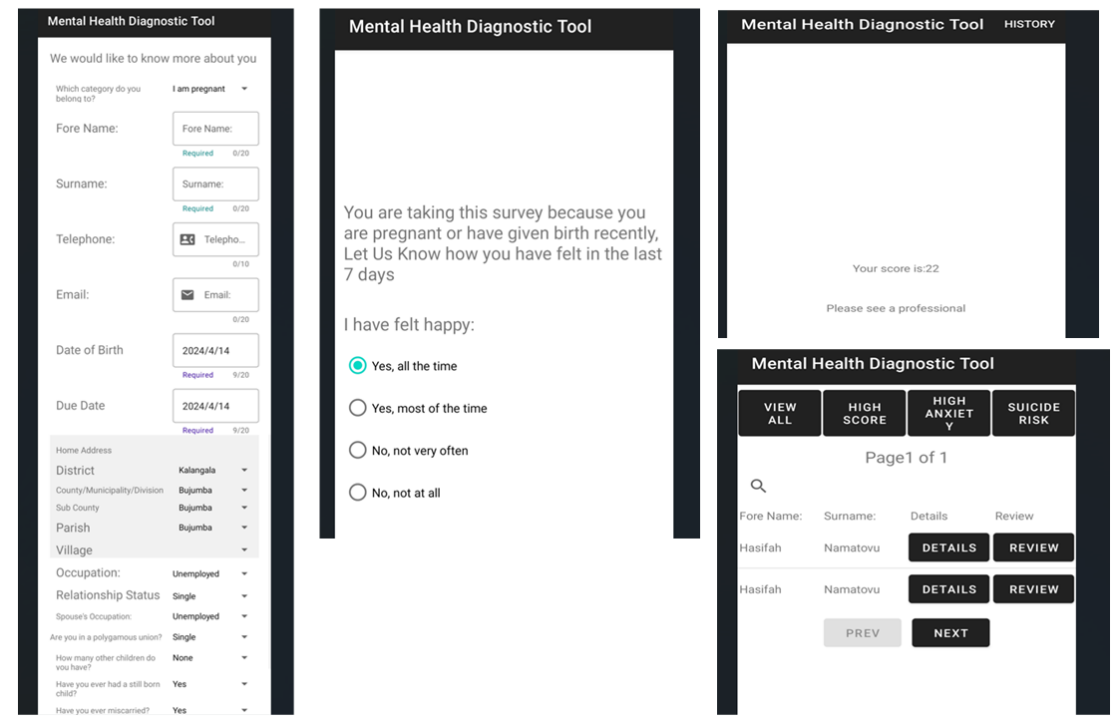
Android implementation of the Edinburgh Postnatal Depression Scale.

The EPDS tool was automated using its standard 10 questions and was implemented via the Android platform installed on the tablet computers as shown in Figure 1. The responses for each of the 10 questions ranged from 0 to 3. For questions 3 and 5‐10, the responses were organized in reverse order (3, 2, 1, and 0). The total score was obtained by adding up the individual scores for all 10 questions. The EPDS was developed to assist health care professionals in identifying mothers experiencing postnatal depression, although it has also been used to screen symptoms of depression in pregnant women [33]. For each of these 10 questions, the woman chose one of four responses reflecting how she felt a week prior to administration of the tool. The responses scored from 0 to 3 are based on the severity of the symptom, with 0 being less severe and 3 being more severe. For safety purposes, a woman who scored 1 or higher on question 10 (indicating suicidal ideation) was immediately referred for further diagnosis since the EPDS is only a screening tool, and not a diagnostic one. Similarly, women who scored 9 or higher were referred for follow-up, as advised by Cox et al [33]. The maximum possible score is 30, with a score of 10 and above indicating possible depression. Women who obtained a score above 13 could possibly be experiencing depressive illness that may vary in severity. To analyze the influence of specific factors on perinatal depression, demographic information was collected as part of the initial sections prior to the administration of the 10 questions of the EPDS.

### Study Participants and Selection

A total of 12,913 prenatal women were recruited for the study from the 7 health facilities. Perinatal services were offered from Monday to Friday, from 8 AM to 1 PM at these facilities. During their visits, the research team was allocated a specific area where they could engage with expectant mothers for depression screening using the Android version of the EPDS. After completing the vital signs assessment, the research assistants randomly selected a respondent for screening. As the EPDS tool was not designed for self-administration, the research assistants would interact with women directly, following the sequence of questions in the scale. This approach was chosen as a significant number of the women were either illiterate or lacked the digital skills required to use a smartphone. To avoid the possibility of a woman discussing their responses, the screening was done in solitude.

### Data Analysis

Data was analyzed using Microsoft Excel. We performed both univariate and bivariate analyses. For the univariate analysis, we established the relative proportions of respondents for each demographic variable. For the bivariate analysis, we examined the relationship between demographic variables and EPDS scores. For categorical variables, we relied on *χ*^2^ tests of independence and risk and odds ratios. We employed a 2-step procedure; first, the *χ*^2^ test of independence was used to determine the demographic variables with the strongest association with the overall EPDS depression categories. Following this, we used the odds ratios to determine which specific values of these variables implied a higher risk for depression. For nonbinary variables such as the spousal or partner employment category, odds ratios were computed as the ratio of odds for respondents in that category to the odds for all other respondents for that variable in a “one versus all” setup. For continuous variables, including the weeks of pregnancy, respondents’ age, and the number of previous children, a correlation analysis was conducted.

### Inclusion Criteria

We included any patient of the antenatal clinics at the respective health facilities who were willing to complete the questionnaire and subsequent EPDS tool.

## Results

### Univariate Analysis

Approximately 66.9% (8633/12,913) of respondents reported being in polygamous unions, 29.7% (n=3838) were first-time mothers, and 49.8% (n=6431) reported having 2 or more previous children. Nearly 11% (1419) of respondents reported having a previously stillborn child, whereas 30.7% (3964) reported experiencing a miscarriage. The average number of children per woman was 1.6. The mothers’ ages ranged from 9-53 (mean 26, SD 5.3) years. The mean EPDS score was 9.8 (SD 3.9), while the median score was 10 (IQR 4). We used the commonly accepted cutoff of 13 points or higher on the EPDS as an indicator of depression [35]. Consequently, we found that 2783 respondents (21.5%; 95% CI 20.8‐22.3) were identified as possibly depressed. These findings have been presented in Table 1.

**Table 1.** Statistical summary of postnatal EPDS data from Kampala health facilities.

Demographics	EPDS^a^ category			Likelihood/odds ratio (95% CI)		Pearson Chi-square		Cramer’s V (effect size)
	Depressed, n (%)	Not depressed, n (%)	Total, n (%)			Chi square (*df*)	*P* value	
Education						76 (4)	*<*.001	0.08 (negligible)
A-level	300 (2.3)	1125 (8.7)	1425 (11)	1.0 (0.8‐1.1)				
Degree/diploma	98 (0.8)	264 (2.0)	362 (2.8)	1.4 (1‐1.7)				
Lower primary/none	289 (2.2)	707 (5.5)	996 (7.7)	1.5 (1.3‐1.8)				
O-level	1534 (11.9)	6403 (49.6)	7937 (61.5)	0.7 (0.7‐0.8)				
P5/higher	562 (4.3)	1631 (12.6)	2193 (17.0)	1.3 (1.2‐1.5)				
Total	2783 (21.5)	10,130 (78.4)	12,913 (100.0)	—^b^				
Respondent occupation						129.63 (4)	*<*.001	0.1 (small)
Formal business owner	200 (1.5)	730 (5.7)	930 (7.2)	1.0 (0.8‐1.2)				
Formal employee	236 (1.8)	338 (2.6)	574 (4.4)	2.7 (2.3‐3.2)				
Informal business owner	370 (2.9)	1808 (14.0)	2178 (16.8)	0.7 (0.6‐0.8)				
Informal employee	223 (1.7)	595 (4.6)	818 (6.3)	1.4 (1.2‐1.6)				
Unemployed	1754 (13.6)	6659 (51.6)	8413 (65.1)	0.9 (0.8‐1.0)				
Total	2783 (21.5)	10,130 (78.4)	12,913 (100.0)	—				
Spouse/partner’s occupation						512 (4)	*<*.001	0.19 (small)
Formal business owner	243 (1.9)	1423 (11.0)	1666 (12.9)	0.6 (0.5‐0.7)				
Formal employee	579 (4.5)	1611 (12.5)	2190 (16.9)	1.4 (1.2‐1.5)				
Informal business owner	443 (3.4)	3132 (24.2)	3575 (27.5)	0.4 (0.4‐0.5)				
Informal employee	829 (6.4)	2605 (20.1)	3434 (26.6)	1.2 (1.1‐1.3)				
Unemployed	689 (5.3)	1359 (10.5)	2048 (15.8)	2.1 (1.9‐2.4)				
Total	2783 (21.5)	10,130 (78.4)	12,913 (100.0)	—				
Relationship status						806.9 (5)	*<*.001	0.25 (small)
Cohabiting	1644 (12.7)	4996 (38.7)	6640 (51.4)	1.5 (1.4‐1.6)				
Formal union	157 (1.2)	530 (4.1)	687 (5.3)	1.1 (0.9‐1.3)				
Separated	159 (1.2)	306 (2.4)	465 (3.6)	1.9 (1.6‐2.4)				
Single	418 (3.2)	730 (5.7)	1148 (8.9)	2.3 (2.0‐2.6)				
Traditional marriage	383 (3.0)	3540 (27.4)	3923 (30.4)	0.3 (0.26‐0.33)				
Widowed	22 (0.2)	28 (0.2)	50 (0.4)	2.9 (1.6‐5.0)				
Total	2783 (21.5)	10,130 (78.4)	12,913 (100.0)	—				
Polygamous union						237.9 (1)	*<*.001	0.13 (small)
Yes	1506 (11.7)	7127 (55.2)	8633 (66.9)	0.5 (0.5‐0.5)				
No	1277 (9.8)	3003 (23.2)	4280 (33.1)	2 (1.8‐2.2)				
Total	2783 (21.5)	10,130 (78.4)	12,913 (100.0)	—				
Previous miscarriage						211.6 (1)	*<*.001	0.12 (small)
Yes	1186 (9.2)	2782 (21.5)	3968 (30.7)	2 (1.8‐2.1)				
No	1597 (12.3)	7348 (57.0)	8945 (69.3)	0.5 (0.5‐0.6)				
Total	2783 (21.5)	10,130 (78.4)	12,913 (100.0)	—				
History of stillbirth						2.99 (1)	*<*.001	0.01 (negligible)
Yes	345 (2.7)	1133 (8.7)	1478 (11.4)	1.1 (1.0‐1.3)				
No	2438 (18.8)	8997 (69.7)	11,435 (88.6)	0.9 (0.8‐1.0)				
Total	2783 (21.5)	10,130 (78.4)	12,913 (100.0)	—				
Health facility						—	—	—
Kasangati HCIII	1 (0.0)	242 (1.9)	243 (1.9)	0.0 (0.0‐0.1)				
Kasubi Kawaala HCIII	304 (2.4)	1091 (8.4)	1395 (10.8)	1.0 (0.9‐1.2)				
Kawempe Mulago Referral Hospital	465 (3.6)	2661 (20.6)	3126 (24.2)	0.6 (0.5‐0.6)				
KCCA^c^ HCIII - Bugolobi	842 (6.5)	538 (4.2)	1380 (10.7)	7.7 (6.9‐8.7)				
Kitebi HCIV	562 (4.3)	646 (5.0)	1208 (9.4)	3.7 (3.3‐4.2)				
Komamboga HCIII	257 (2.0)	3306 (25.6)	3563 (27.6)	0.2 (0.2‐0.2)				
Mengo Kisenyi HCIV	352 (2.7)	1646 (12.7)	1998 (15.4)	0.7 (0.7‐0.8)				
Total	2783 (21.5)	10,130 (78.4)	12,913 (100.0)	—				

a EPDS: Edinburgh Postnatal Depression Scale.

b Not applicable.

c KCCA: Kampala Capital City Authority.

#### Education Level

The most common education level was O-level, accounting for 61.2% (n=7937), followed by higher primary school (P5 or higher) at 17% (n=2193), A-level education at 11% (n=1425), and lower primary school at approximately 7.7% (n=996), as demonstrated in Table 1. Lastly, tertiary level education (degree or diploma), accounted for 2.8% (n=362). In Uganda, O-level corresponds to the Ordinary Level Certificate of Education, comparable to the UK’s General Certificate of Education, whereas A-Level corresponds to the Advanced Certificate of Education.

#### Respondents’ Employment Status

The most common employment category among respondents was unemployed, accounting for 65.1% (8413/12,913). This was followed by 16.8% (n=2178) informal business owners, 7.2% (n=930) formal business owners, 7.2% informal employees (n=930), and 4.4% (n=574) formal employees. Formal business owners refer to individuals who own formally registered businesses, whereas informal business owners include market vendors, hawkers, and small-scale farmers who own informal businesses. Informal employees are those engaged in short-term work on a daily or weekly basis, without a formal contract, whereas formal employees are employed on a long-term basis with well-stipulated contracts, similar to the majority of white-collar workers.

#### Employment Status of Respondents’ Spouse or Partner

The most common employment category of respondents’ spouses or partners was informal business owners, accounting for 27.5% (n=3575) of respondents, followed by those with informally employed spouses or partners representing 26.6% (n=3434) of respondents. Nearly 16.9% (n=2190) of respondents had spouses or partners who were formal employees, whereas 15.8% (n=2048) of respondents had unemployed spouses, and 12.9% (n=1666) of respondents had spouses who were formal business owners.

#### Relationship Status

The most common relationship status among respondents, accounting for 51.4% (n=6640) of respondents, was cohabiting representing partners living together with no formal or traditional marriage, followed by traditional marriages representing 30.4% (n=3923) of respondents. Single women comprised 8.9% (n=1148) of respondents, while those in formal unions (church, mosque, or civil weddings) comprised 5.3% (n=687) of respondents, and women who had separated from their spouses or partners constituted 3.6% (n=465) of respondents. Lastly, widowed women comprised 0.4% (n=50) of respondents.

#### Polygamous Unions

This variable refers to whether the respondent was in a relationship with a partner who had one or more additional sexual partners. A majority (n=8633, 66.9%) of respondents reported that they were in polygamous relationships.

#### Previous Miscarriage

This variable refers to whether a woman had previously experienced a miscarriage. We found that nearly 30.7% (n=3968) of respondents reported a history of miscarriage, in agreement with previously published studies [36].

#### Previous Stillbirth

This variable refers to whether the respondent had previously given birth to a stillborn child. We found that a total of 11.4% (n=1478) of respondents reported a stillborn child. These results align closely with previously reported data [37].

### Bivariate Analysis

For the bivariate analysis, we initially used the *χ*^2^ test to determine which demographic features had a strong influence on the EPDS classification. We then calculated the odds ratio for each feature to understand its effects better. Table 1 includes odds ratios for various health facilities for completeness; however, we excluded the hospital as a variable for determining depression risk. This decision was due to variable data collection periods across different facilities, indicating that the sample sizes may not accurately reflect the typical volume of clients for each facility, making cross-facility comparisons statistically unreliable.

Among the remaining categories (education level, respondents’ occupation, spouse or partners’ occupation, polygamous union, previous miscarriage, and stillbirth), the respondents’ relationship status had the largest effect on their EPDS score (*χ*^2^=806.9, *P*<.001; Cramer’s V=0.25). The effect size was determined using Cramer’s V and *df* as per the method described by Kim [38]. As seen in Table 1, widowed women had the highest odds ratio for depression and were nearly 3 times more likely to be depressed compared to nonwidowed women. Conversely, women in traditional marriages were the least likely to report depression compared to other groups.

For quantitative variables, we found a negative correlation between respondents’ age and the total depression score (*r*=−0.11, *P*<.001). Similarly, there was a negative correlation between total depression and number of previous children *(r*=−0.11, *P*<.001). A weak negative correlation was also observed between weeks of gestation and the total depression score (*r*=−0.03,* P*=.002).

## Discussion

### Principal Findings

We found an overall prevalence of 21.5% (95% CI 20.8%-22.3%). We could not locate previous studies from Uganda that focused specifically on prenatal or antenatal mothers and used the EPDS; this prevalence falls within the range of previous studies in similar regions [39,40]. Relationship status was the most important determinant of depression. The group most likely to display depressive symptoms was widowed women. Broadly, women who referred to themselves as single, separated, or widowed displayed a significantly higher propensity for depression compared to those who were partnered. This outcome is in agreement with previous studies [41] and is not surprising given that spousal or partner support has been widely recognized as an important factor [40,42,43].

The correlation analysis revealed a weak negative relationship between maternal depression during pregnancy and the number of children. Although we found no previous studies specifically investigating this correlation, there is indirect corroboration from studies suggesting that women with more children reported lower levels of depression [44,45]. However, the direction of causality may be reversed, as individuals prone to depression are also less likely to have more children [46]. Furthermore, the correlation analysis revealed a weak negative association between age and antenatal depression, in agreement with some previous studies [47]. However, other studies have reported contrary findings [48]. This may be attributed to age being correlated with latent variables such as income and parental experience, which may vary across different cohorts. For instance, we found a very strong positive correlation between age and the number of children implying that one of these variables may be confounding. We also observed a negative correlation between the depression scores and weeks of gestation. This is in agreement with some previous studies that have noted a reduction in depression as pregnancy progresses [49]. However, the association in our study was too weak to merit extensive discussion.

### Limitations of the Study

A key limitation of this study is its reliance on indirect parameters such as spousal or partner employment categories, rather than directly measuring household income and spousal support. This limitation arises because the demographic data were originally collected as preliminary input for creating user profiles in the EPDS Android app. Important missing parameters that could provide deeper insights into the woman’s depressive state include social support, intimate partner violence, early and unplanned pregnancies, physical and verbal abuse, self-esteem issues, emotional abuse, and physical health parameters concerning the pregnancy and the woman’s overall health. These factors could be explored in follow-up studies with smaller, more focused cohorts. However, this study provides a valuable statistical overview of prenatal depression in Kampala, Uganda.

### Implications of the Study

These findings imply that with sufficient data, empirical approaches could be developed to identify individuals at risk of depression. For instance, demographic markers such as respondents’ relationship statuses have a strong influence on the EPDS depression categories and could be used to create risk profiles or automate interventions.

### Conclusion

Perinatal screening for depression is not commonly performed during and after pregnancy, despite being emphasized by the World Health Organization. This study revealed that women whose spouses or partners were engaged in some form of employment, especially those in informal business ownership were less likely to experience depression.

## Abbreviations

EPDS: Edinburgh Postnatal Depression Scale
KCCA: Kampala Capital City Authority

## References

[1] Poppert Cordts KM, Wilson AC, Riley AR (2017). More than mental health: parent physical health and early childhood behavior problems. Physiol Behav.

[2] All about providing emotional support to women during pregnancy. Apollo Cradle and Children’s Hospital.

[3] Akesson B (2008). Addressing the psychosocial needs of pregnant women affected by war: program approaches and program gaps. Refuge.

[4] Sarkar NDP, Bardaji A, Peeters Grietens K, Bunders-Aelen J, Baingana F, Criel B (2018). The social nature of perceived illness representations of perinatal depression in rural Uganda. Int J Environ Res Public Health.

[5] Lund C, Town C (2016). Maternal depression. https://d1wqtxts1xzle7.cloudfront.net/81141598/Crick-Lund-Reading-Pack-libre.pdf?1645431793=&response-content-disposition=inline%3B+filename%3DMaternal_Depression.pdf&Expires=1734715766&Signature=CNtej2tyYCqE7SC2gDHLk6ZTVH6T2PcbnJ7yaxZ4~PTEP0TIOg9Tv6HDj698o0jY-ik2dLUj~FLnnZhThVxPL0cRZ0TqZpWHb2ha3fDyFxDTm-zXL-bV3XVKoyr30p8PuT-iBfNSEjK5YMmTn8mtEwxPiGUhLL6cf~3S87Wg0WRsN2k0b9abPquiPtAXxnA2x6egSF61UIo1bcDLGqcyt5OsDaVCaqlPk4fTDdWb~lGk86yBEvLI2HymeBPEvuZAAezTfKXepnnL6hEx1~wzTE6pvr2nUK7j4f2yz-WTjYZqa-YIa8hqQ42sCAqXKHyflG9GME~OnQu2BfRCWj5xbQ__&Key-Pair-Id=APKAJLOHF5GGSLRBV4ZA.

[6] Tol WA, Ebrecht B, Aiyo R (2018). Maternal mental health priorities, help-seeking behaviors, and resources in post-conflict settings: a qualitative study in eastern Uganda. BMC Psychiatry.

[7] Baron EC, Hanlon C, Mall S (2016). Maternal mental health in primary care in five low- and middle-income countries: a situational analysis. BMC Health Serv Res.

[8] Muzik M, Borovska S (2010). Perinatal depression: implications for child mental health. Ment Health Fam Med.

[9] Nwoke CN, Awosoga OA, McDonald S, Bonifacio GT, Leung BMY (2023). African immigrant mothers’ views of perinatal mental health and acceptability of perinatal mental health screening: quantitative cross-sectional survey study. JMIR Form Res.

[10] Fisher J, Cabral de Mello M, Patel V (2012). Prevalence and determinants of common perinatal mental disorders in women in low- and lower-middle-income countries: a systematic review. Bull World Health Organ.

[11] Nemoda Z, Szyf M (2017). Epigenetic alterations and prenatal maternal depression. Birth Defects Res.

[12] Field J (2009). Good for your soul? Adult learning and mental well‐being. Int J Lifelong Educ.

[13] de Bruijn A, van Bakel HJA, van Baar AL (2009). Sex differences in the relation between prenatal maternal emotional complaints and child outcome. Early Hum Dev.

[14] Atukunda P, Muhoozi GKM, Westerberg AC, Iversen PO (2019). Nutrition, hygiene and stimulation education for impoverished mothers in rural Uganda: effect on maternal depression symptoms and their associations to child development outcomes. Nutrients.

[15] Krohn H, Guintivano J, Frische R, Steed J, Rackers H, Meltzer-Brody S (2022). App-based ecological momentary assessment to enhance clinical care for postpartum depression: pilot acceptability study. JMIR Form Res.

[16] Shi P, Ren H, Li H, Dai Q (2018). Maternal depression and suicide at immediate prenatal and early postpartum periods and psychosocial risk factors. Psychiatry Res.

[17] Patel V, Saxena S, Lund C (2018). The Lancet Commission on global mental health and sustainable development. Lancet.

[18] Cox EQ, Sowa NA, Meltzer-Brody SE, Gaynes BN (2016). The perinatal depression treatment cascade. J Clin Psychiatry.

[19] Nakku JEM, Nakasi G, Mirembe F (2006). Postpartum major depression at six weeks in primary health care: prevalence and associated factors. Afr Health Sci.

[20] Ayano G, Demelash S, Yohannes Z (2021). Misdiagnosis, detection rate, and associated factors of severe psychiatric disorders in specialized psychiatry centers in Ethiopia. Ann Gen Psychiatry.

[21] Altamura AC, Goikolea JM (2008). Differential diagnoses and management strategies in patients with schizophrenia and bipolar disorder. Neuropsychiatr Dis Treat.

[22] Nasrallah HA (2015). Consequences of misdiagnosis: inaccurate treatment and poor patient outcomes in bipolar disorder. J Clin Psychiatry.

[23] Nakku JEM, Okello ES, Kizza D (2016). Perinatal mental health care in a rural African district, Uganda: a qualitative study of barriers, facilitators and needs. BMC Health Serv Res.

[24] Muhwezi WW, Agren H, Musisi S (2007). Detection of major depression in Ugandan primary health care settings using simple questions from a subjective well-being (SWB) subscale. Soc Psychiatry Psychiatr Epidemiol.

[25] Rush JA (2000). Handbook of Psychiatric Measures.

[26] Spies G, Kader K, Kidd M (2009). Validity of the K-10 in detecting DSM-IV-defined depression and anxiety disorders among HIV-infected individuals. AIDS Care.

[27] Baggaley RF, Ganaba R, Filippi V (2007). Detecting depression after pregnancy: the validity of the K10 and K6 in Burkina Faso. Trop Med Int Health.

[28] Kaaya SF, Fawzi MCS, Mbwambo JK, Lee B, Msamanga GI, Fawzi W (2002). Validity of the Hopkins Symptom Checklist-25 amongst HIV-positive pregnant women in Tanzania. Acta Psychiatr Scand.

[29] Derogatis LR, Lipman RS, Rickels K, Uhlenhuth EH, Covi L (1974). The Hopkins Symptom Checklist (HSCL): a self-report symptom inventory. Behav Sci.

[30] Kushniruk AW, Myers K, Borycki EM, Kannry J (2009). Exploring the relationship between training and usability: a study of the impact of usability testing on improving training and system deployment. Stud Health Technol Inform.

[31] Chibanda D, Mangezi W, Tshimanga M (2010). Validation of the Edinburgh Postnatal Depression Scale among women in a high HIV prevalence area in urban Zimbabwe. Arch Womens Ment Health.

[32] Sheehan DV, Lecrubier Y, Sheehan KH (1998). The MINI-International Neuropsychiatric Interview (M.I.N.I.): the development and validation of a structured diagnostic psychiatric interview for DSM-IV and ICD-10. J Clin Psychiatry.

[33] Cox JL, Holden JM, Sagovsky R (1987). Detection of postnatal depression. Development of the 10-item Edinburgh Postnatal Depression Scale. Br J Psychiatry.

[34] (2018). National health facility master list. Ministry of Health Republic of Uganda.

[35] Matthey S (2008). Using the Edinburgh Postnatal Depression Scale to screen for anxiety disorders. Depress Anxiety.

[36] Asiki G, Baisley K, Newton R (2015). Adverse pregnancy outcomes in rural Uganda (1996-2013): trends and associated factors from serial cross sectional surveys. BMC Pregnancy Childbirth.

[37] Moyer CA, Kolars CK, Oppong SA, Bakari A, Bell A, Busingye P (2016). Predictors of stillbirths and neonatal deaths in rural western Uganda. Intl J Gynecology Obste.

[38] Kim HY (2017). Statistical notes for clinical researchers: chi-squared test and Fisher’s exact test. Restor Dent Endod.

[39] Rahman A, Iqbal Z, Harrington R (2003). Life events, social support and depression in childbirth: perspectives from a rural community in the developing world. Psychol Med.

[40] Umuziga MP, Gishoma D, Hynie M, Nyirazinyoye L (2022). Antenatal depressive symptoms in Rwanda: rates, risk factors, and social support. BMC Pregnancy Childbirth.

[41] Yin X, Sun N, Jiang N (2021). Prevalence and associated factors of antenatal depression: systematic reviews and meta-analyses. Clin Psychol Rev.

[42] Lee EJ, Lee JY, Lee SJ, Yu SE (2020). Influence of self-esteem and spouse support on prenatal depression in pregnant women. J Korean Soc Matern Child Health.

[43] Hu Y, Wang Y, Wen S (2019). Association between social and family support and antenatal depression: a hospital-based study in Chengdu, China. BMC Pregnancy Childbirth.

[44] Yang H, Zheng X, Zhou R, Shen Z, Huang X (2020). Fertility behavior and depression among women: evidence from China. Front Psychol.

[45] Kravdal Ø, Grundy E, Skirbekk V (2017). Fertility history and use of antidepressant medication in late mid-life: a register-based analysis of Norwegian women and men. Aging Ment Health.

[46] Golovina K, Elovainio M, Hakulinen C (2023). Association between depression and the likelihood of having children: a nationwide register study in Finland. Am J Obstet Gynecol.

[47] Li X, Gao R, Dai X (2020). The association between symptoms of depression during pregnancy and low birth weight: a prospective study. BMC Pregnancy Childbirth.

[48] Muraca GM, Joseph KS (2014). The association between maternal age and depression. J Obstet Gynaecol Can.

[49] Lee AM, Lam SK, Sze Mun Lau SM, Chong CSY, Chui HW, Fong DYT (2007). Prevalence, course, and risk factors for antenatal anxiety and depression. Obstet Gynecol.

